# Cost-effectiveness of anti-retropulsive devices varies according to the locations of proximal ureteral stones: a retrospective cohort study

**DOI:** 10.1186/s12894-022-00995-9

**Published:** 2022-03-24

**Authors:** Weisong Wu, Jiaqiao Zhang, Rixiati Yi, Xianmiu Li, Xiao Yu

**Affiliations:** Department of Urology, Tongji Hospital, Tongji Medical College, Huazhong University of Science and Technology, Wuhan, China

**Keywords:** Ureteral calculi, Anti-retropulsive device, Cost-effective, Ureteroscopy, Stone-free

## Abstract

**Background:**

Anti-retropulsive devices are often used to prevent stone migration in the treatment of proximal ureteral calculi. They are helpful. However, in the meantime, they also add extra expenses. This study was carried out to investigate the best criteria for treating proximal ureteral stones with anti-retropulsive devices.

**Methods:**

Data from all patients who underwent ureteroscopic holmium: YAG laser lithotripsy for solitary upper ureteral stones in 2018 were collected. Patients who encountered stone retropulsion during the process of inserting the ureteroscope were excluded. Patients were divided into either group URS or group URS + ARD depending on whether the anti-retropulsive device was used. Then, the stone-free rate, expenses and other criteria were compared between groups according to stone location. Stone-free was defined as no stones present.

**Results:**

For stones located ≤ 30 mm from the ureteropelvic junction (UPJ), the stone-free rates for the URS group were 80% and 80% at one day and one month after the operation, respectively. Those for the URS + ARD group were 71.4% and 78.6% at one day and one month, respectively. For stones located 31–90 mm from the UPJ, the stone-free rates were 84.7% and 84.7% for the URS group and 89.6% and 95.5% for the URS + ARD group at one day and one month, respectively. A statistically significant difference occurred at one month. For stones located > 90 mm from the UPJ, the two groups were both stone free. In the URS + ARD group, expenses were higher. In addition, the mean diameter of residual stones derived from stones located at 31–90 mm from the UPJ was statistically smaller, and 4 of 7 residual stones passed spontaneously within one month, which was obviously more than that in other locations and the URS group. Other outcomes, including operation time and postoperative stay, showed no significant difference between the groups.

**Conclusion:**

Anti-retropulsive devices are indeed helpful, but they might be cost-effective for stones located solely in the middle part of the upper ureter, not for those too close to or far from the ureteropelvic junction.

**Supplementary Information:**

The online version contains supplementary material available at 10.1186/s12894-022-00995-9.

## Introduction

Ureteral calculi, as one of the most common diseases in urology, have been prevalent for years, often presenting acute attacks and leading to renal colic. Quick pass leaves almost no damage. However, due to the existence of the three anatomical physiological narrow places of the ureter, not all stones could pass, especially those large ones. When stones fail to pass spontaneously and medical therapy or active stone removal is indicated, timely surgery should be considered to protect renal function. Shock wave lithotripsy and endourological procedures, including semirigid ureteroscopy (URS), flexible ureteroscopy and percutaneous nephrolithotomy, are common methods [1]. Of them, URS has gained popularity due to efficiency and relatively low requirements of equipment and skills and something else [2]. Although URS is recommended as an effective treatment for ureteral stones in all locations, stone-free rates (SFRs) differ greatly in different locations: approximately 85% for proximal ureter locations, 90% for mid-ureter locations and 94% for distal ureter locations [3]. Retropulsion, defined as unintended migration of stone or debris back into the kidney, leads to most failure in the proximal ureter locations and results in a lower SFR [4]. Many measures have been taken to reduce retropulsion, such as the Trendelenburg position and anti-retropulsive devices (ARDs) [5–7].

In processing upper ureteral calculi, ARDs are often used, which have various shapes but work similarly by fixing the stones and preventing upmigration [8]. URS with ARD indeed improves the stone-free rate but also brings an extra expense. In theory, it will not be cost-effective for all upper ureteral stones. Prior studies demonstrated that the usage of ARD would be cost-effective if the retropulsion rate was greater than 6.3% [9]. Nevertheless, clinical experience indicates that the closer the stones are from the ureteropelvic junction (UPJ), the higher the rate of upmigration back into the renal space might be. In addition, not all retropulsions could be successfully prevented by ARD. What was worse, some debris managed to pass the ARD and finally left in the kidney, which also diminished its priority. Therefore, we conducted this study hoping to investigate the best optimal occasion for URS in treating proximal ureteral stones with ARD.

## Methods

A retrospective cohort study of proximal ureteral stone treatment with and without ARD was conducted. Patients with solitary upper ureteral stones from January 2018 to December 2018 were divided into either the URS group or the URS + ARD group by whether ARD was used. Then, patients were further analysed according to the distance of stones from the UPJ. Patients who encountered stone retropulsion during the process of inserting the ureteroscope met our exclusion criteria. Stone diagnosis was confirmed by multiple modalities, principally by noncontrast-enhanced computed tomography (NCCT). All stones were disintegrated by 8/9.8 F semirigid ureteroscope with holmium:YAG laser 1–1.5 J, 15–20 Hz in our department. ARD referred to N-Trap, Cook Medical (Additional file 1: Figure S1). A JJ stent was placed on all patients, fixed with pigtail coils two to four weeks after the operation and followed up with for one year. Pre, intra- and postoperative outcomes were compared between groups.

All clinical data were collected from inpatient medical records, and follow-up results were obtained from outpatient medical records and telephone surveys focused on prognosis. Dilation up to the renal pelvis, central and peripheral calices dilated, and already result in parenchyma thin were defined as mild, moderate and severe grade of hydronephrosis, respectively [10]. Expenses were obtained from inpatient billing. Stone free meant no stone left was judged by kidney-ureter-bladder (KUB) at one day and one month after the operation. Stone residual meant that there was stone present, no matter the size. The distance from the UPJ was defined as the vertical distance to the maximum plane of the stone. Based on frequency distribution and clinical experience, the ureter was divided into three parts, ≤ 30 mm, 31–90 mm, and > 90 mm.

We used SPSS v.26 to process the data. Numerical variables were tested for normality and are presented as the mean ± SD. Then, an independent-samples t test was performed. χ^2^ or Fisher's exact test was used to analyse the counting data. *p* < 0.05 was recorded as statistically significant.

## Results

As seen in Table 1, for patients whose stones were located ≤ 30 mm from the UPJ, all baseline data between the URS group and URS + ARD group were similar. The two groups had comparable demographic data, including sex, age, BMI, and similar stone characteristics, including length, width, burden, density and hydronephrosis grade. Patients in the groups had almost the same operation time and SFR at one day and one month. Regardless of whether the ARD was used, SFRs were not high enough, 80% for the URS group and 71.4% for the URS + ARD group at one day and 80% and 78.6% at one month. Surprisingly, expense between groups also showed no significant difference.

**Table 1:** Baseline characteristics of patients whose stones were located ≤ 30 mm from the UPJ

	Type of procedure		*p* value
	URS	URS + ARD	
All patients. no	25	14	
Gender, no. Male/Female	12/13	7/7	1.000
Age, years, mean ± SD, median [range]	49.4 ± 15.9, 48 [22–73]	46.1 ± 11.8, 44.5 [27–65]	0.504
BMI, mean ± SD, median [range]	23.7 ± 2.9, 23.5 [19.5–31.2]	24.4 ± 3.3, 25.7 [18.0–29.4]	0.478
Hydronephrosis grade, no. Mild/Moderate/Severe	15/7/3	11/3/0	0.317
Stone laterality (L/R), no	10/15	6/8	1.000
Stone size, mean ± SD median [range]			
Length (mm)	10.2 ± 4.0, 10 [5–20]	11.6 ± 4.2, 11 [4–18]	0.298
Width(mm)	6.6 ± 2.1, 6 [4–10]	8.2 ± 3.3, 7.5 [3–15]	0.069
Burden (mm^2^)^a^	57.5 ± 38.2, 47.1 [15.7–157.0]	82.6 ± 55.3, 65.9 [7.1–176.6]	0.102
Density (HU)	1129.4 ± 327.5, 1119, [467–1849]	1268.8 ± 301.0, 1332.5 [619–1643]	0.254
Operation time (min), mean ± SD, median [range]	40.1 ± 19.0, 42 [12–94]	44.5 ± 25.3, 42.5 [18–82]	0.648
Intraoperation complications	0	0	–
Postoperative stay (days), mean ± SD, median [range]	4.0 ± 1.7, 4 [2–8]	3.2 ± 2.1, 2 [1–7]	0.240
Post-fever, no	0	1	0.359
Post-sepsis, no	0	1	0.359
Total expense (¥), mean ± SD, median [range]	22,693.6 ± 11,528.6, 18,644.5 [11693.0 ± 54,848.0]	23,839.6 ± 5046.9, 23,295.7 [17096.0 ± 33,675.0]	0.729
SFR (%)			
One-day	80.0	71.4	0.696
One-month	80.0	78.6	1.000

^a^Stones Burden = Length* width*0.25*3.14

For patients whose stones were located 31–90 mm from the UPJ, the SFRs were 84.7% for the URS group and 89.6% for the URS + ARD group at one day and 84.7% and 95.5% at one month, as presented in Table 2. A statistically significant difference occurred at one month. A higher SFR was observed when ARD was used, despite a larger stone length and burden. The Expense of the URS + ARD group was obviously higher than that of the URS group. Other data, including postoperative stay and complications, showed no significant difference. No significant reduction in operative time was observed.

**Table 2:** Baseline characteristics of patients whose stones were located 31–90 mm from the UPJ

	Type of procedure		*p* value
	URS	URS + ARD	
All patients. no	72	67	
Gender, no. Male/Female	48/24	53/14	0.128
Age, years, mean ± SD, median [range]	52.0 ± 15.1, 51 [27–88]	47.5 ± 12.9, 48 [22–76]	0.065
BMI, mean ± SD, median [range]	24.9 ± 3.1, 24.9 [17.6–33.0]	25.3 ± 4.4, 24.5 [14.2–37.3]	0.589
Hydronephrosis grade, no. Mild/Moderate/Severe	45/17/10	31/28/8	0.070
Stone laterality (L/R), no	10/15	6/8	1.000
Stone size, mean ± SD median [range]			
Length (mm)	10.3 ± 3.4, 10 [5–25]	12.5 ± 5.5, 11 [5–36]	**0.006**
Width (mm)	6.8 ± 2.0, 7 [3–15]	7.4 ± 2.1, 7 [4–11]	0.101
Burden (mm^2^)	58.0 ± 33.4, 51.8 [14.1–212.0]	78.3 ± 54.3, 64.4 [15.7–310.9]	**0.010**
Density (HU)	1207.2 ± 307.8, 1239 [420–1815]	1239.8 ± 319.7, 1323 [451–1738]	0.617
Operation time (min), mean ± SD, median [range]	40.2 ± 18.4, 35.5 [10–84]	44.5 ± 27.8, 36.5 [14–150]	0.304
Intraoperation complications	0	0	–
Postoperative stay (days), mean ± SD, median [range]	4.3 ± 1.3, 4 [1–8]	4.2 ± 2.1, 4 [2–13]	0.710
Post-fever, no	3	3	1.000
Post-sepsis, no	3	1	0.621
Total expense (¥), mean ± SD, median [range]	20,303.1 ± 5832.4, 20,071.3 [10040.0 ± 45,145.0]	26,204.7 ± 12,106.7, 24,280.1 [5912.0 ± 87,707.0]	**0.001**
SFR (%)			
One-day	84.7	89.6	0.455
One-month	84.7	95.5	**0.047**

Bold fonts to highlight the statisitical significance

For patients whose stones were located > 90 mm from the UPJ, as shown in Table 3, none of the stones or debris migrated back into the kidney and resulted in 100% SFRs in either group. Only expense showed a significant difference between the two groups. Similar to Table 2, significantly higher expenditure was observed in the URS + ARD group.

**Table 3:** Baseline characteristics of patients whose stones were located > 90 mm from the UPJ

	Type of procedure		*p* value
	URS	URS + ARD	
All patients. no	22	14	
Gender, no. Male/Female	19/3	13/1	1.000
Age, years, mean ± SD, median [range]	51.0 ± 14.4, 50.5 [27–86]	46.7 ± 12.3, 50.5 [25–64]	0.390
BMI, mean ± SD, median [range]	24.8 ± 3.5, 24.0 [15.6–31.2]	24.0 ± 1.9, 23.7 [21.5–26.7]	0.482
Hydronephrosis grade, no. Mild/Moderate/Severe	11/6/5	9/4/1	0.457
Stone laterality (L/R), no	10/15	6/8	1.000
Stone size, mean ± SD median [range]			
Length (mm)	12.2 ± 3.9, 12 [5–20]	11.0 ± 3.3, 12 [4–15]	0.351
Width (mm)	7.5 ± 2.3, 8 [3–11]	6.5 ± 1.6, 6 [3–9]	0.163
Burden (mm^2^)	75.4 ± 35.6, 75.4 [11.8–141.3]	58.4 ± 24.2, 63.6 [9.4–91.9]	0.127
Density (HU)	1266.3 ± 266.8, 1330.5 [775–1630]	1149.9 ± 237.6, 1157 [782–1530]	0.300
Operation time (min), mean ± SD, median [range]	38.1 ± 19.8, 35 [14–90]	46.3 ± 13.1, 44 [25–67]	0.210
Intraoperation complications	0	0	–
Postoperative stay (days), mean ± SD, median [range]	3.8 ± 1.5, 3 [2–7]	3.7 ± 1.4, 3.5 [1–6]	0.841
Post-fever, no	0	0	–
Post-sepsis, no	0	0	–
Total expense (¥), mean ± SD, median [range]	19,389.6 ± 4841.9, 19,365.4 [12,566.0 ± 27,265.0]	24,127.0 ± 4938.1, 24,621.7 [16,076.0 ± 32,041.0]	**0.011**
SFR (%)			
One-day	100.0	100.0	–
One-month	100.0	100.0	–

Bold fonts to highlight the statisitical significance

Table 4 shows that the mean diameters of the residual stones located < 30 mm from the UPJ in the two groups were similar, but those located 31–90 mm from the UPJ were significantly larger in the URS group than in the URS + ARD group. In addition, the proportion (4/7) of residual stones derived from stones located 31–90 mm from the UPJ in the URS + ARD group that passed spontaneously within one month was higher than that in the other groups.

**Table 4:** Prognosis of residual stones within one month according to stone locations

	Type of procedure		*p* value
	URS	URS + ARD	
*Stones from UPJ (mm)*			
≤ 30			
Residual stone^a^, No	5	4	
Diameter (mm)	8.8 ± 2.9	6.0 ± 2.2	0.158
Spontaneous pass, No	0	1	0.444
31–90			
Residual stone^a^, No	11	7	
Diameter (mm)	8.0 ± 3.1	4.9 ± 2.2	**0.035**
Spontaneous pass, No	0	4	**0.011**
> 90			
Residual stones^a^, No	0	0	–

Bold fonts to highlight the statisitical significance

^a^Evaluated at the time of one day after operation

## Discussion

Due to the high stone-free rate and minimal invasiveness, ureteroscopy has a steep rising trend of usage [2, 11]. Indeed, ureteroscopy shows effectiveness in ureteral calculi in all locations. However, hindered by retropulsion, the SFR for proximal stones is not as high as that for other locations. This is because the semirigid ureteroscope is difficult to access above the level of the renal pelvis. ARD works by fixing the stone and avoiding upmigration, and it has been proven in many articles that it succeeds in bringing a higher stone-free rate [12]. However, the extra charge should not be ignored. Since all other outcomes, including operation time, postoperative stay, complications and so on, were comparable between groups, making it cost-effective shows great clinical significance. Our study indicates that it is helpful only in the middle part of the upper ureteral calculi.

Consistent with the majority of previous papers, our data support the conclusion that ARD is helpful in pursuing a higher SFR [4]. Without ARD, URS showed only a 72% stone-free rate, as reported by Wang et al. in 2017 [13]. However, our study showed that the high SFR did not appear until one month later and only in stones from the UPJ 31–90 mm. For stones located ≤ 30 mm or > 90 mm from the UPJ, ARD did not play a crucial role in improving SFR. In detail, ARD was not helpful enough to prevent stones that were too close to the UPJ from migrating. The same phenomenon was found in a study comparing Ho:YAG laser and pneumatic lithotripters [14]. ARD was used in all patients. However, the raw data revealed that the residual stones of ≤ 4 mm in diameter for stones closer than 2 cm to the UPJ was 64% in the laser group, which was even lower in the pneumatic group. On the other hand, ARD was not necessary for stones far from the UPJ because there was no stone that managed to move back into the kidney.

In total, ten fragments and one whole stone in the URS + ARD group were found left in the kidney after the operation. The latter was caused by ARD failing to fix the stone. These large stones were mainly derived from stones located ≤ 30 mm from the UPJ, and they were too large to pass through the ureter spontaneously within one month. It was easy to explain it. Fragments passed the ARD through the crack between ARD and the ureter wall. The closer the stone is from the UPJ, the larger the lumen diameter, and the higher the incidence of retropulsion. In addition, ureteral stones are always accompanied by dilation of the above ureter. On the other hand, each kind of ARD has its own working radius and mesh size. The largest open diameter of the NTrap used in this study was 15 mm. It was large enough for most positions. However, the uppermost ureter could not completely block the greatly dilated lumen. Meanwhile, the chance for urologists to relocate the moved stones was very small among those stones that were too close to the UPJ, so it was easy to result in a large size of stone left and consequently a low stone-free rate. For stones from UPJ > 90 mm, both modalities achieved a 100% SFR. Even without ARD, there were ample opportunities for urologists to relocate the migrated stone, if there was.

Sorts of efforts have been made to reduce or avoid retropulsion. Patel RM et al. conducted an in vitro trial and reported that ureteral stone gravity worked well in the proper position [6]. They suggested increasing the patients incline angel. Anterograde irrigation, Trendelenburg position, negative pressure, "Guidewire-Coil"-Technique and so on have also been published as useful to reduce retropulsion [5, 15–17]. However, these methods currently could hardly provide an enormous promotion similar to ARD or are held back by additional requirements.

There are many kinds of anti-retropulsive devices. They mostly work in a similar way. They block the ureter or fix the stone. The subtle difference lies in convenience, cost, materials, etc. The majority of prior studies reached unanimous conclusions regardless of the types of ARDs, such as N-Trap and Stone Cone [18]. Devices are similar in terms of preventing proximal stone migration [19]. Therefore, we have reason to believe that the conclusion is applicable to other products.

Unexpectedly, expenses did not show a marked difference between the two groups in Table 1. However, we found that the median expense in the URS + ARD group was approximately 4,000 ¥ more than that in the URS group, which was the price of the ARD. The reason for this was probably incidental errors. The number of cases was small. The difference in the stone-free rate at Day one was not apparent, possibly because of the criteria that none of the debris could be present. Many other congeneric articles defined fragments of ≤ 4 mm or 2 mm as stonefree, which were reported as clinically insignificant stones [20].

In summary, it was not cost-effective to use ARD in all proximal stones. Flexible ureteroscopy, extracorporeal shockwave lithotripsy or percutaneous nephrolithotomy might be an alternative for uppermost ureteral stones [21, 22]. For proximal stones far from the UPJ, there was no need to use the ARD. For proximal ureteral stones located in the middle, ARD was strongly recommended.

The main limitations of the study were its retrospective design and low sample size in a single centre. Only one device was tested. It was not enough. In further research, we will include more kinds of ARD. Furthermore, we had no direct data about the ureteral lumen diameter. The proportions of impacted stone and stone compositions were also unknown. The distance of stones from the UPJ was replaced by the vertical dimension, which might lead to bias. Moreover, SFR judged by KUB was not precise enough. Finally, cost analysis is very superficial, and a more convincing evaluation is needed.

## Conclusion

Anti-retropulsive devices are indeed helpful in preventing stones from migrating and achieving a high stone-free rate, and the effect in the middle part of the proximal ureter is significant, but they might not be cost-effective for proximal ureteral stones that are too close to or far from the UPJ.

## Supplementary Information

**Additional file 1: Figure S1.** Appearance of the used N-trap.

## Data Availability

The datasets generated and/or analysed during the current study are not publicly available due to limitations of ethical approval involving the patient data and anonymity but are available from the corresponding author upon reasonable request.

## Abbreviations

URS: Ureteroscopy
ARD: Anti-retropulsive device
UPJ: Ureteropelvic junction
SFR: Stone-free rate
KUB: Plain film of kidney-ureter-bladder
