# Supplementary figures and images for: Cost-effectiveness of anti-retropulsive devices varies according to the locations of proximal ureteral stones: a retrospective cohort study
Source: BMC Urol. 2022 Mar 24;22:43. doi: 10.1186/s12894-022-00995-9 (PMC8952225; doi:10.1186/s12894-022-00995-9)

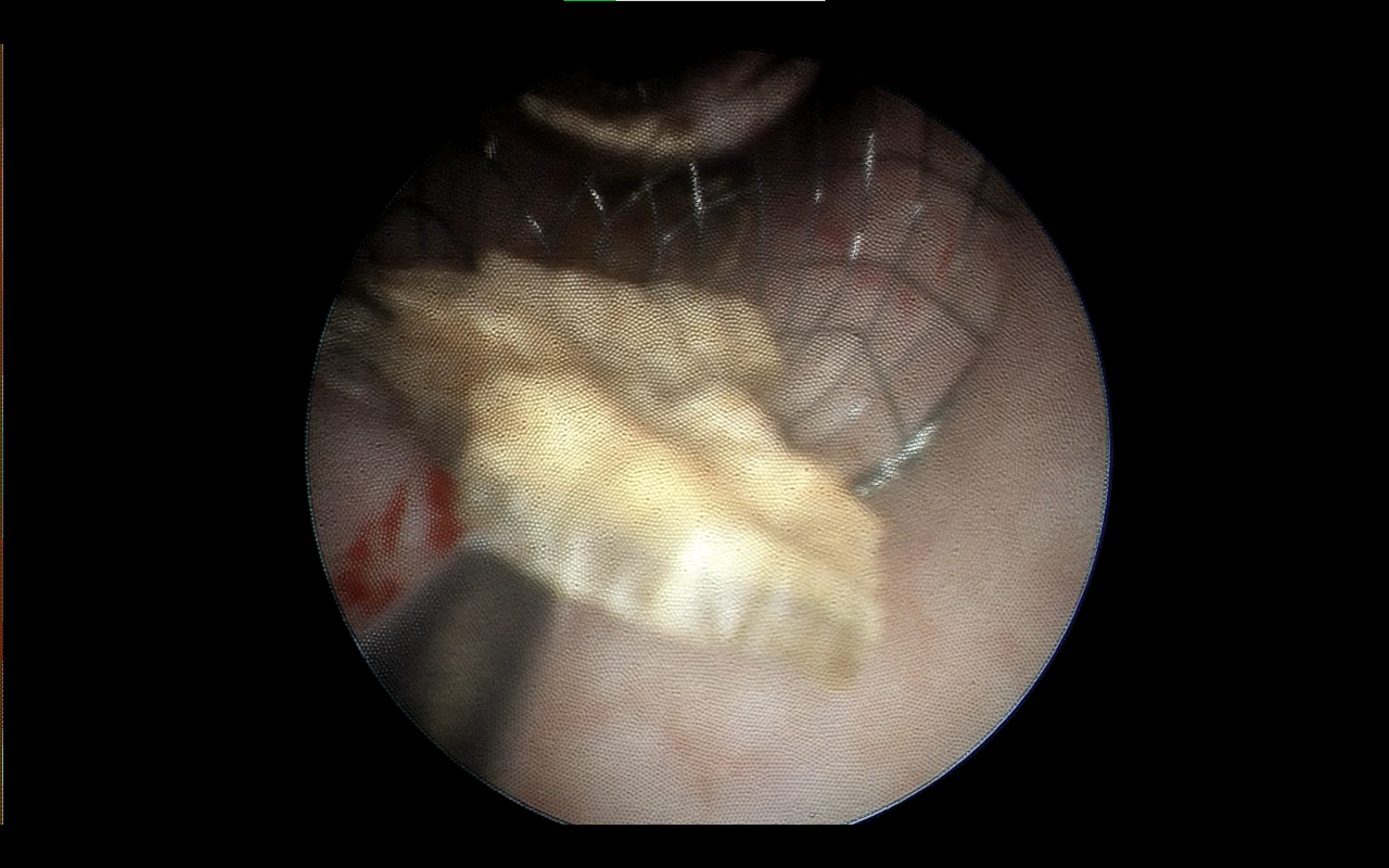

Supplement: Supplementary file 1 — Additional file 1: Figure S1. Appearance of the used N-trap. [file 12894_2022_995_MOESM1_ESM.png]
